# The International Hospital Congress at Chicago

**Published:** 1893-06-10

**Authors:** 


### The Hospital

An Institutional Journal of

**Science, Medicine, Nursing, and Philanthropy.**

*For Hospitals, Asylums, Infirmaries, Dispensaries, Medical Practitioners, Students, Nurses, and the Charitable Public.*

Vol. XIV.—No. 350.] June 10, 1893.

### THE INTERNATIONAL HOSPITAL CONGRESS AT CHICAGO.

Chicago is making a valiant attempt to settle all the difficulties of the Universe. Shakespeare tells us that "Our doubts are traitors, and oft make us lose the good we would by fearing to attempt." Chicago and her Exhibition are troubled by no such traitorous inward tremblings. There appears to be nothing which they "fear to attempt" in the City of the West, not even the solution of the ten thousand times unsolved problems of the hospitals.

What will the Congress on Hospitals do? We know it will talk. But its talk, we believe, is to be strictly limited. Even the readers of papers are to be allowed no more than fifteen minutes each; and the speakers, we presume, will not be indulged with more than a fifth part of that liberal allowance. If rush and concentration can accomplish anything, the Chicago Congress should show us some wondrous results.

The Congress will not open until June 15th, and we are, therefore, left at present to the vague possibilities of conjecture. There are elements, however, in the constitution of the Congress which give a certain promise of worthy results. First and chief among these is the President. Dr. J. S. Billings is a man of world-wide fame—at any rate, in the medical profession. A great reputation requires great deeds for its sustenance. By a happy ordination of Providence the greatest names wither and die if those who have built them up fail to sustain them by continuous and advancing greatness. Let us remind our readers, and Dr. Billings himself, of some of those achievements which have raised him high above the level of the average medical man. We do not go back to the rather ancient history of the American War. Considerations of space bid us fix attention on one or two more recent events associated with the name and fame of Dr. Billings.

The Johns Hopkins Hospital, at Baltimore, was described by our Special Commissioner in 1891 as "the most interesting hospital in the world." That hospital is, to a very great extent, the creation of Dr. J. S. Billings. The very reason which makes so many institutions and buildings uninteresting, that is their newness, is the chief point of interest in the Johns Hopkins Hospital. Newness ordinarily suggests rawness, crudity, incompleteness. The exact opposite of all these is true of the Johns Hopkins Hospital. Rawness and crudity are the last ideas one would associate with it. Completeness is its one distinguishing mark. There, on that spot at Baltimore, are concentrated all that science, all that art, all that experience and the deepest devotion to humanity could suggest for the relief of pain and the restoration of health; and there also are to be found the perfection of scientific teaching and every conceivable means and facility for the philosophic advancement of medical science and technique. What medical science and art can do for humanity is done at the Johns Hopkins Hospital, and it is done to a very large extent under the authority and the inspiration of Dr. J. S. Billings.

But Dr. Billings has not been merely a leader and guide of medical men and specialists. He has gone out into the broader arena of common mankind, and there held up the torch of physiological and medical truth for the guidance of the commonest and the meanest. At this moment, as we write, we hold in our hand an ordinary evening newspaper, the *Wash-ing'on Evening Star*, which devotes no less than six entire columns to an expository lecture by Dr. Billings, wherein he sets forth with admirable lucidity, for the instruction of the stupidest, the broad principles of life saving by means of preventive medicine. Dr. Billings is a Washington man; and he is reasonably proud of his city. But, like a true physician, he sees that he can never be as truly proud of his city as he ought to be until every preventible death is prevented, and every man, woman, and child is raised to the highest possible level of happiness and health. Such an ideal as this is worthy of a great physician. The true physician does not live for guineas and a sounding name; he lives that he may bring the blessings of his beneficent art to the aid and physical salvation of every necessitous human being in the world. Such an ideal is something to live for; a lower is only worthy of sordid selfishness and mean medical hacks.

The main points of Dr. Billings' scheme may be put into a paragraph, and they are so admirably practical that all intelligent readers will be grateful to us for publishing them. To begin with, the author modestly calls his scheme a "Sanitary League." The editor of the *Washington Evening Star*, with the true journalist's instinct, christens it "A League Against Death." Dr. Billings offers himself as the leader of a sanitary army, and the army is to consist of every man, woman, and child of sense in Washington. Inasmuch as every man, woman, and child is exposed to the assaults of the forces of death, so every man, woman, and child should unite to constitute the army of "The League Against Death." The League, when constituted, will consist of a grand committee, with departmental and local committees, and associated workers of every class in every part of the city. "Do not wait for an invitation to join," says Dr. Billings, "but send in your names and volunteer. We must obtain the co operation of the clergy, of the working men's organisations, of the Mission School for Educating Housekeepers, of those who are engaged in the work of educating our coloured people, and special committees for these various objects are recommended. There is also to be a committee of ladies on sanitary housekeeping." There will, in a word, be five departmental committees, one for rules and regulations, one for statistics and reports, one for printing and auditing, a promoter's committee, and a committee on co-operation. The League, by means of its various departments, will deal with the water supply, sewage disposal, infectious hospitals, a public disinfecting plant, the collection and disposal of garbage, the disposal of the dead, public wash and bath houses, slaughter-houses and markets, dairies and other sources of food supply, and the sanitary conditions of public school buildings. "One of the essential features of the League," says Dr. Billings, "is to deal with details. It is through the local committees that it is proposed to collect information." House to house inspection will be systematically carried out, and the district authorities will furnish leaflets full of condensed practical information to those housekeepers who do not attend lectures or read books. In a word, to quote Dr. Billings once more, "If all these committees do their work, and do it well: if the information gained is used to the best advantage, the result will be to improve the health of the City, to do away with certain causes of disease and death, and to make Washington a safer place of residence for intelligent people."

The man who had the intellect to conceive this large scheme, and so to propound it to an audience of the general public as to make them one and all instantaneously decide to take it up with full tide Transatlantic enthusiasm, is the President of the International Congress of Hospitals and Hospital Authorities. Has the distinguished President formulated as practical a scheme as this for the consideration of the Hospital authorities of the world? Or is he depending upon the readers of departmental papers to produce and round up a scheme by their disjointed individual efforts? If the President is prepared with a complete scheme of hospital development and administration, the product of his own fertile and experienced brain, the prospects of good are brighter than a congress of experts usually warrants us in looking for; but if all we are to expect of the Congress is a series of unconnected papers by persons with a tendency to special crotchets and faddism, we shall not hope for many wise and permanent solutions of the difficult hospital problems of the day.

